# Knowledge About COVID-19 Among Adults in China: Cross-sectional Online Survey

**DOI:** 10.2196/26940

**Published:** 2021-04-29

**Authors:** Fengyun Yu, Pascal Geldsetzer, Anne Meierkord, Juntao Yang, Qiushi Chen, Lirui Jiao, Nadeem E Abou-Arraj, An Pan, Chen Wang, Till Bärnighausen, Simiao Chen

**Affiliations:** 1 Department of Industrial Engineering Tsinghua University Beijing China; 2 Heidelberg Institute of Global Health Faculty of Medicine and University Hospital Heidelberg University Heidelberg Germany; 3 Division of Primary Care and Population Health Department of Medicine Stanford University School of Medicine Stanford, CA United States; 4 Faculty of Medicine University of Southampton Southampton United Kingdom; 5 State Key Laboratory of Medical Molecular Biology Institute of Basic Medical Sciences Chinese Academy of Medical Sciences and Peking Union Medical College Beijing China; 6 The Harold and Inge Marcus Department of Industrial and Manufacturing Engineering The Pennsylvania State University University Park, PA United States; 7 Reed College Portland, OR United States; 8 Department of Medicine Stanford University School of Medicine Stanford, CA United States; 9 School of Public Health Tongji Medical College Huazhong University of Science and Technology Wuhan China; 10 Chinese Academy of Medical Sciences and Peking Union Medical College Beijing China; 11 National Clinical Research Center for Respiratory Diseases Beijing China; 12 Department of Pulmonary and Critical Care Medicine Center of Respiratory Medicine China–Japan Friendship Hospital Beijing China

**Keywords:** COVID-19, knowledge, perception, risk, public health, China, cross-sectional, survey

## Abstract

**Background:**

A detailed understanding of the public’s knowledge and perceptions of COVID-19 could inform governments’ public health actions in response to the pandemic.

**Objective:**

The aim of this study was to determine the knowledge and perceptions of COVID-19 among adults in China and its variation among provinces and by sociodemographic characteristics.

**Methods:**

Between May 8 and June 8, 2020, we conducted a cross-sectional online survey among adults in China who were registered with the private survey company KuRunData. We set a target sample size of 10,000 adults, aiming to sample 300-360 adults from each province in China. Participants were asked 25 questions that tested their knowledge about COVID-19, including measures to prevent infection, common symptoms, and recommended care-seeking behavior. We disaggregated responses by age; sex; education; province; household income; rural–urban residency; and whether or not a participant had a family member, friend, or acquaintance who they know to have been infected with SARS-CoV-2. All analyses used survey sampling weights.

**Results:**

There were 5079 men and 4921 women who completed the questionnaire and were included in the analysis. Out of 25 knowledge questions, participants answered a mean and median of 21.4 (95% CI 21.3-21.4) and 22 (IQR 20-23) questions correctly, respectively. A total of 83.4% (95% CI 82.7%-84.1%) of participants answered four-fifths or more of the questions correctly. For at least one of four ineffective prevention measures (using a hand dryer, regular nasal irrigation, gargling mouthwash, and taking antibiotics), 68.9% (95% CI 68.0%-69.8%) of participants answered that it was an effective method to prevent a SARS-CoV-2 infection. Although knowledge overall was similar across provinces, the percent of participants who answered the question on recommended care-seeking behavior correctly varied from 47.0% (95% CI 41.4%-52.7%) in Tibet to 87.5% (95% CI 84.1%-91.0%) in Beijing. Within provinces, participants who were male, were middle-aged, were residing in urban areas, and had higher household income tended to answer a higher proportion of the knowledge questions correctly.

**Conclusions:**

This online study of individuals across China suggests that the majority of the population has good knowledge of COVID-19. However, a substantial proportion still holds misconceptions or incorrect beliefs about prevention methods and recommended health care–seeking behaviors, especially in rural areas and some less wealthy provinces in Western China. This study can inform the development of tailored public health policies and promotion campaigns by identifying knowledge areas for which misconceptions are comparatively common and provinces that have relatively low knowledge.

## Introduction

COVID-19 has taken a large toll on public health and economic growth worldwide [1-4]. Assessing the perception and knowledge among the public during infectious disease outbreaks is essential to inform public health campaigns. Research has shown that governmental policies can have a substantial impact on community transmission of SARS-CoV-2 [5-8]. It is likely that the more detailed an understanding governments have of their population’s knowledge and perceptions of COVID-19, the more effectively they can design policies to contain COVID-19 in their population, whether this is on the national, regional, or local level.

A cross-sectional survey conducted in late February 2020 that assessed the public’s perceptions of COVID-19 in the United States and the United Kingdom found that a considerable proportion of adults had misconceptions about infection prevention methods and care-seeking behaviors [9]. For example, over a third of survey participants selected at least one of the following options when asked whether they are effective prevention measures: using a hand dryer, rinsing your nose with saline, taking antibiotics, or gargling with mouthwash. Reasons for these false beliefs are unclear but could be different for populations in East Asian countries like China or Singapore, which were affected by the severe acute respiratory syndrome outbreak in 2002-2004.

Assessing the population’s perceptions and knowledge of COVID-19 is not only essential to understanding and comparing different behaviors and policy decisions retrospectively but also vital for informing postlockdown policies since it will be crucial that people follow infection prevention methods as they start to interact more [10,11]. As of November 2020, China appears to have been successful in containing the spread of COVID-19 and has reported low case numbers [12]. However, further waves of COVID-19 may emerge in parts of China over the coming months. Therefore, collecting data about the knowledge and perception of COVID-19 across China is imperative.

Several studies have assessed the perception of COVID-19 within China. However, they have either focused on specific subgroups, such as pregnant women [13] or patients with mental health disorders [14], or assessed risk perception within particular contexts such as tourism [15]. To our knowledge, our study is the first large-scale survey that assessed COVID-19 perception and knowledge among the public in all provinces of China. This study aims to inform Chinese policy makers on the knowledge and perceptions of their population with regard to COVID-19 to facilitate effective policy design during future waves of the pandemic.

## Methods

### Sampling Process

The survey was implemented by KuRunData, an online private survey company that maintains a database of potential survey participants and delivers surveys. KuRunData recruits members through its own platform [16], partnerships with other websites, and encouraging registered members to recruit new members through the popular mobile app Wechat Mini. KuRunData verifies that members have access to mobile phones and the internet, and are capable of navigating online surveys. For this study, we used KuRunData to sample 300-360 participants in each of China’s 31 provincial-level administrative units, with the total sample size goal being 10,000 adults. Potential participants were unable to access the questionnaire as soon as this sample size goal was reached. Within each province, KuRunData aimed to sample a proportion of participants that was reflective of the demographic composition of the province’s population (as per the 2019 China Statistical Yearbook [17]) by sex and urban-rural residence. Adults in the survey pool were invited to participate in the survey by KuRunData’s own platform. They were informed that they would receive ¥5 (US $0.77) for completing the questionnaire. Before filling in the questionnaire, participants had to provide their informed written consent with signature confirmation. The informed consent page described the project’s background and purpose, the possible risks, the payment after completing the questionnaire, and the confidentiality of information and records. To be able to access the questionnaire, the participants must have read the informed consent description for at least 15 seconds and self-declared understanding of the purpose and risks of the study before signing. The survey was administered between May 8 and June 8, 2020.

### Questionnaire

The questionnaire was built in the KuRunData platform and had 25 questions partitioned into the following sections: introduction, perceived risk of death from COVID-19, mode of transmission of COVID-19, recognizing and acting upon an infection, sociodemographic characteristics, and specific questions about possible misconceptions or falsehoods on COVID-19 prevention and symptoms that were drawn from the World Health Organization’s “myth busters” website [18]. The questionnaire was written in Standard Chinese and is shown in Text A1 in Multimedia Appendix 1. Participants had to answer a question to reach the next question. Numerical entry questions did not allow for nonsensical inputs (eg, percentage questions were restricted to inputs between 0 and 100).

### Data Quality Checks

Three types of data quality checks were performed. First, we verified the time taken to complete the questionnaire and excluded participants who took less than 2 minutes to complete the questionnaire under the assumption that these participants did not read the questions. Second, we plotted the distribution of the time taken to complete the questionnaire. If some respondents used random clicking to complete the questionnaire as fast as possible, then a bimodal distribution in the time taken to complete the survey might be expected (with one study population clicking as quickly as possible and one reading the questions). Third, participants were asked whether they looked up any answers online and, if so, for which questions. Those who self-reported having looked up the answer online for a particular question were excluded from the analysis for that question in the supplementary analyses shown in Multimedia Appendix 1.

### Data Analysis

We excluded participants who answered less than half of the questions in the questionnaire. All analyses used sampling weights to account for the complex survey design. The sampling weights were the inverse of the probability of selecting participants given the following variables: gender, rural versus urban residence, and province. These probabilities were calculated using population counts from the 2019 China Statistical Yearbook within each province. For binary and categorical response options, we computed the percentage of participants who selected each response to summarize the survey findings. For binomial proportions, we constructed two-sided 95% CIs using the Wilson score interval. In addition, we computed a total score for participants, which consists of the number of COVID-19 knowledge questions that were answered correctly. We henceforth refer to this score as the *overall knowledge score*. To examine how knowledge and perceptions varied by participants’ characteristics, we used ordinary least squares regression to regress this overall score and the response to each question onto age (10-year age group); sex; educational attainment; province; rural versus urban residence; vocation; household income; and whether or not a participant had a family member, friend, or acquaintance who they knew to have been infected with SARS-CoV-2. All regressions included only one of these variables plus a binary indicator for each province (province-level fixed effects). We show regression results that we additionally adjusted for 10-year age group and sex in Multimedia Appendix 1.

### Ethics

This research was considered to not involve human participants by the institutional review board of the Heidelberg University Hospital because all authors only had access to deidentified data.

## Results

### Sample Characteristics

A total of 14,493 adults agreed to take the online survey. After excluding participants who did not complete the whole survey or who took less than 2 minutes to complete the questionnaire, 10,000 participants (all of whom completed all survey questions) were included in the analysis. There was no evidence of a bimodal distribution in the time taken to complete the questionnaire (Multimedia Appendix 1 Figure A1). A total of 3643 participants reported looking up the answer online on a median of 2 questions (IQR 1-3).

There were 5079 males and 4921 females from 31 provinces that completed the questionnaire. Their sociodemographic characteristics are shown in Table 1. Around one-tenth of the 10,000 participants (n=900, 9.0%) were aged 18 or 19 years, 16.5% (n=1645) were aged 20-29 years, 19.0% (n=1895) were aged 30-39 years, and 16.8% (n=1675) were 60 years or older. A total 37.3% (n=3733) of the participants had received high school or technical secondary school education, and one-third (n=3369, 33.7%) had completed an undergraduate degree. Only 4.4% (n=438) and 4.8% (n=475) of participants had never been to school or had been to elementary school only, respectively. The majority of participants (n=5935, 59.3%) lived in urban areas. The number of participants per province ranged from 300 to 360. About half (n=5007, 47.0%) of participants reported to have an annual total household income between ¥60,000 (US $9180) and ¥119,999 (US $18,360).

**Table 1:** Sample characteristics.

Characteristic			Proportion of participants (weighted^a^), %		Participants (not weighted), n (%)		Population of China, %^b^
**Sex**							
	Female	56.5		4921 (49.2)		48.8	
**Age group (years)**							
	<20	10.6		900 (9.0)		6.9	
	20-29	16.9		1645 (16.5)		20.8	
	30-39	18.4		1895 (19.0)		18.2	
	40-49	18.5		1890 (18.9)		22.1	
	50-59	17.9		1820 (18.2)		16.5	
	>60	17.7		1675 (16.8)		15.4	
**Education**							
	Never been to school	4.1		438 (4.4)		5.4	
	Elementary school	4.3		475 (4.8)		25.3	
	Middle school	16.3		1779 (17.8)		37.8	
	High school/technical secondary school	35.7		3733 (37.3)		17.6	
	College/undergraduate	37.3		3369 (33.7)		13.4	
	Graduate and above	2.2		206 (2.0)		0.6	
**Ethnicity**							
	Han	95.1		9381 (93.8)		95.0	
	Man	0.5		149 (1.5)		0.7	
	Hui	0.1		109 (1.1)		0.8	
	Zang	1.6		103 (1.0)		0.5	
	Zhuang	1.5		152 (1.5)		1.2	
	Other	1.1		106 (1.1)		1.8	
**Province of current residence**							
	Anhui	4.4		360 (3.6)		4.5	
	Beijing	1.8		360 (3.6)		1.5	
	Chongqing	2.3		360 (3.6)		2.2	
	Fujian	2.8		300 (3.0)		2.8	
	Gansu	1.8		300 (3.0)		1.9	
	Guangdong	8.6		360 (3.6)		8.1	
	Guangxi	3.3		300 (3.0)		3.5	
	Guizhou	2.4		300 (3.0)		2.6	
	Hainan	0.7		300 (3.0)		0.7	
	Hebei	5.3		360 (3.6)		5.4	
	Heilongjiang	2.7		300 (3.0)		2.7	
	Henan	6.3		360 (3.6)		6.9	
	Hubei	4.2		360 (3.6)		4.2	
	Hunan	4.8		300 (3.0)		4.9	
	Jiangsu	6.1		360 (3.6)		5.8	
	Jiangxi	3.2		300 (3.0)		3.3	
	Jilin	1.9		300 (3.0)		1.9	
	Liaoning	3.3		340 (3.4)		3.1	
	Neimengol	1.8		300 (3.0)		1.8	
	Ningxia	0.5		300 (3.0)		0.5	
	Qinghai	0.4		300 (3.0)		0.4	
	Shaanxi	2.7		360 (3.6)		2.8	
	Shandong	7.3		360 (3.6)		7.2	
	Shanghai	2.0		300 (3.0)		1.7	
	Shanxi	2.6		300 (3.0)		2.7	
	Sichuan	5.7		360 (3.6)		6.0	
	Tianjin	1.3		360 (3.6)		1.1	
	Tibet	1.7		300 (3.0)		0.2	
	Xinjiang	0.2		300 (3.0)		1.8	
	Yunnan	3.2		300 (3.0)		3.5	
	Zhejiang	4.3		360 (3.6)		4.1	
**Rural–urban residency**							
	Urban	69.5		5935 (59.3)		59.6	
**Works as a health care provider**							
	No	96.0		9597 (96.0)		99.0	
	Nurse	0.5		55 (0.6)		0.3	
	Physician	0.8		84 (0.8)		0.5	
	Community health worker	1.5		157 (1.6)		<0.1	
	Pharmacist	0.2		17 (0.2)		<0.1	
	Other health care provider	1.0		90 (0.9)		0.1	
**Annual household income, ¥ (US $)**							
	<30,000 (3835)	5.7		560 (5.6)		—^c^	
	30,000-59,999 (3835-7670)	14.1		1670 (16.7)		—	
	60,000-89,999 (7670-11,505)	20.4		2303 (23.0)		—	
	90,000-119,999 (11,506-15,341)	25.0		2704 (24.0)		—	
	120,000-149,999 (15,341-19,176)	15.5		1211 (12.1)		—	
	150,000-199,999 (19,175-25,568)	12.2		974 (9.7)		—	
	≥200,000 (25,568)	7.2		578 (5.8)		—	

^a^Weighted using survey sampling weights.

^b^As per the 2019 China Statistical Yearbook [17].

^c^Data not available.

### Overall Knowledge Score

**Figure 1:**
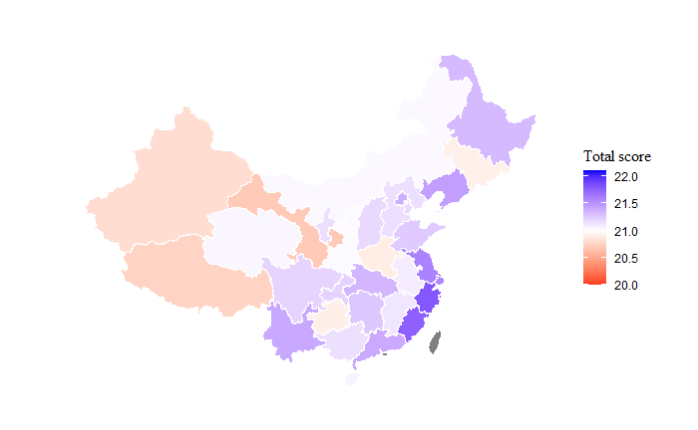
Map showing the mean overall knowledge score by province.

A total 83.4% (95% CI 82.7%-84.1%) of participants answered 80% or more (ie, 20 or more out of 25 questions) of the questions correctly, and almost all (98.4%, 95% CI 98.1%-98.6%) participants answered more than 60% of the questions correctly (Figure 1). The mean and median overall knowledge score was 21.4 (95% CI 21.3-21.4) and 22 (IQR 20-23), respectively. The distribution of the overall knowledge score is shown in Figure A2 in Multimedia Appendix 1.

Participants residing in the eastern provinces tended to have marginally higher overall knowledge scores. For instance, the mean knowledge score in the eastern province of Fujian was 21.9 (95% CI 21.6-22.1), whereas it was 20.9 (95% CI 20.6-21.2) in the western province of Gansu.

### Perceived Risk of Death From a SARS-CoV-2 Infection

Survey participants’ median estimate of the infection-fatality rate of COVID-19 was 3.2% (IQR 1.0%-3.6%; Table 2). When asked to estimate the percentage of patients infected with the common flu who die from the flu, participants’ median response was 0.75% (IQR 0.10%-1.00%). Almost all (96.4%, 95% CI 96.0%-96.8%) participants identified that older adults were the age group most likely to die from COVID-19.

**Table 2:** Summary of survey findings.

Survey question and response^a^				Proportion or median estimate^b^
**Perceived risk of death from a SARS-CoV-2 infection**				
	“**What percent of individuals infected with the new coronavirus experience a fatal disease course?” (%), median (IQR)**			
		Continuous variable	3.2 (1.0-3.6)	
	“**When they have been infected, what age groups are most likely to die from the illness caused by the new coronavirus?” % (95% CI)**			
		Children	25.0 (24.2-25.9)	
		Young adults	12.0 (11.4-12.6)	
		Older adults	96.4 (96.1-96.8)	
	“**Are those with other health problems more likely to die from an infection with the new coronavirus disease than those without any other health problems?” % (95% CI)**			
		Yes	92.5 (92.0-93.0)	
	“**What percent of people who get infected with the common flu end up dying from the common flu?” (%), median (IQR)**			
		Continuous variable	0.75 (0.10-1.00)	
**Transmission of SARS-CoV-2, % (95% CI)**				
	“**Only older adults can become infected with the new coronavirus.”**			
		False	98.3 (98.0-98.5)	
	“**Is there currently a vaccine available that protects against infection with the new coronavirus?”**			
		No	78.9 (78.1-79.7)	
	“**Which of the following actions help prevent catching an infection with the new coronavirus?”**			
		Selected all of the following: avoiding touching eyes, nose, and mouth with unwashed hands; washing your hands; and avoiding close physical contact with people who are sick	86.3 (85.6-86.9)	
		Selected at least one of the following: using a hand dryer, regularly rinsing your nose with saline, taking antibiotics, and gargling mouthwash	68.9 (68.0-69.8)	
	“**Consistently wearing a face mask is highly effective in protecting you from getting infected with the new coronavirus.”**			
		True	89.5 (88.9-90.1)	
	“**What is the main way in which people are currently getting infected with the new coronavirus?”**			
		Droplets of saliva that land in the mouths or noses of people who are nearby when an infected person sneezes or coughs	82.2 (81.5-83.0)	
**Symptoms of COVID-19 and recommended health care–seeking behavior, % (95% CI)**				
	“**What are common signs or symptoms of an infection with the new coronavirus?”**			
		Nose bleeds	9.7 (9.1-10.2)	
		Cough	98.2 (97.9-98.5)	
		Fever	99.5 (99.4-99.6)	
		Skin rash	8.7 (8.2-9.3)	
		Constipation	7.4 (6.9-7.9)	
		Shortness of breath	91.5 (91.0-92.1)	
		Frequent urination	4.5 (4.1-4.9)	
	“**If you have a fever and new persistent cough that started today, what would you do?” % (95% CI)**			
		Go directly to a hospital	36.9 (35.9-37.8)	
		Call the official hotline	38.6 (37.7-39.6)	
		Continue my daily routine	10.9 (10.3-11.6)	

^a^Response options are grouped to summarize categorical variables into a dichotomous measure.

^b^For dichotomous outcomes, data are expressed as percentage with correct response (95% CI). For continuous outcomes, data are expressed as median (IQR).

### Transmission of SARS-CoV-2

A total 98.3% (95% CI 98.0%-98.5%) of participants correctly identified that COVID-19 does not only afflict older adults. When asked to identify the primary mode of transmission from a list of multiple choices, 82.2% (95% CI 81.5%-83.0%) of participants correctly selected a description of droplet transmission. A total 89.5% (95% CI 88.9%-90.1%) of participants answered that wearing a face mask was highly effective in protecting against infection, and 86.3% (95% CI 85.6%-86.9%) of participants selected correct behavioral prevention measures. However, 68.9% (95% CI 68.0%-69.8%) of participants answered that at least one of the following measures helps prevent SARS-CoV-2 infection: using a hand dryer, regular nasal irrigation, gargling mouthwash, and taking antibiotics.

### Symptoms of COVID-19 and Recommended Health Care–Seeking Behavior

Most participants correctly identified the common symptoms of COVID-19: fever (99.5% answered correctly, 95% CI 99.4%-99.6%), cough (98.2% answered correctly, 95% CI 97.9%-98.5%), and shortness of breath (91.5% answered correctly, 95% CI 91.0%-92.1%). Conversely, when asked about symptoms that are not characteristic of COVID-19, participants correctly answered that these were not expected symptoms of the disease: relatively few participants believed that nose bleeds (9.7%, 95% CI 9.1%-10.2%), skin rash (8.7%, 95% CI 8.2%-9.3%), constipation (7.4%, 95% CI 6.9%-7.9%), or frequent urination (4.5%, 95% CI 4.1%-4.9%) were symptoms of COVID-19.

When asked what they should do if they developed new symptoms of fever and cough, 36.9% (95% CI 35.9%-37.8%) stated they would go directly to a hospital or contact their community health worker or other official contact person, 38.6% (95% CI 37.7%-39.6%) said they would call the official hotline, and 10.9% (95% CI 10.3%-11.6%) reported that they would continue their usual daily routines.

### Variation in Knowledge and Perceptions of COVID-19 by Sociodemographic Characteristics

Participants who were male, were middle-aged (we found the highest knowledge score among the age group 40-49 years), were living in an urban area, and had a higher annual household income were more likely to answer knowledge questions correctly (Table 3). On average, participants living in urban areas answered an additional 1.33 (95% CI 1.22-1.44) questions correctly compared to participants living in rural areas. Men answered an additional 0.42 (95% CI 0.32-0.52) questions correctly compared to women.

**Figure 2:**
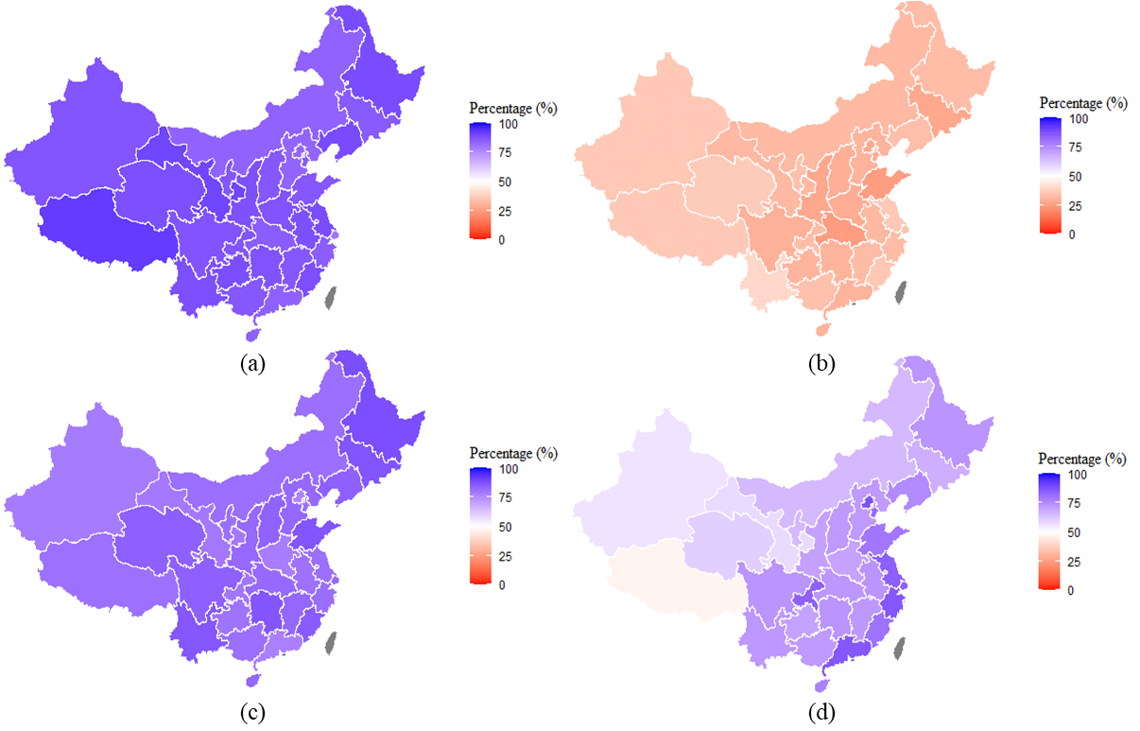
The proportion of the population by province with correct responses to questions about (a) prevention methods, (b) common misconceptions, (c) transmission channels, and (d) recommended actions after infection.

Although there was relatively little variation in knowledge between provinces for prevention methods, common misconceptions, and the main mode of SARS-CoV-2 transmission (Figure 2), a higher proportion of participants in eastern coastal provinces answered the question on recommended care-seeking behavior correctly than in western inland provinces. The range in the proportion of correct responses for this question varied from 47.0% (95% CI 41.4%-52.7%) in Tibet to 87.5% (95% CI 84.1%-91.0%) in Beijing.

**Table 3:** Variation in the overall knowledge score by sociodemographic characteristics.^a^

Characteristic			Absolute difference in the number of questions that were answered correctly (95% CI)		*P* value
**Sex**					
	Male	0 (reference)		N/A^b^	
	Female	–0.42 (–0.52 to –0.32)		<.001	
**Age (years)**					
	18-19	0 (reference)		N/A	
	20-29	0.68 (0.46 to 0.9)		<.001	
	30-39	1.03 (0.8 to 1.25)		<.001	
	40-49	1.17 (0.95 to 1.38)		<.001	
	50-59	0.68 (0.46 to 0.9)		<.001	
	>60	0.75 (0.53 to 0.97)		<.001	
**Education**					
	Never been to school	0 (reference)		N/A	
	Elementary school	–0.18 (–0.53 to 0.16)		.30	
	Middle school	–0.36 (–0.63 to –0.08)		.01	
	High school/technical secondary school	–0.47 (–0.72 to –0.21)		<.001	
	College/undergraduate	0.20 (–0.05 to 0.46)		.12	
	Graduate and above	–0.11 (–0.52 to 0.3)		.60	
**Place of residence**					
	Rural	0 (reference)		N/A	
	Urban	1.33 (1.22 to 1.44)		<.001	
**Works as a health care provider**					
	No	0 (reference)		N/A	
	Nurse	–0.34 (–1.12 to 0.45)		.40	
	Physician	–0.59 (–1.48 to 0.3)		.20	
	Community health worker	–1.13 (–1.75 to –0.51)		<.001	
	Pharmacist	0.06 (–1.44 to 1.57)		.93	
	Other health care provider	–0.94 (–1.58 to –0.29)		.004	
**Annual household income, ¥ (US $)**					
	<30,000 (3835)	0 (reference)		N/A	
	30,000-59,999 (3835-7670)	0.76 (0.45 to 1.08)		<.001	
	60,000-89,999 (7670-11,505)	0.92 (0.62 to 1.22)		<.001	
	90,000-119,999 (11,506-15,341)	1.27 (0.97 to 1.57)		<.001	
	120,000-149,999 (15,341-19,176)	1.59 (1.27 to 1.9)		<.001	
	150,000-199,999 (19,175-25,568)	1.61 (1.29 to 1.93)		<.001	
	≥200,000 (25,568)	1.56 (1.21 to 1.92)		<.001	
**Knows someone with a confirmed SARS-CoV-2 infection**					
	No	0 (reference)		N/A	
	Self	2.09 (–3.04 to 7.23)		.43	
	Family member	–2.66 (–9.26 to 3.93)		.43	
	Neighbor	–0.76 (–2.76 to 1.24)		.45	
	Coworker	0.97 (–0.56 to 2.51)		.21	
	Friend	–2.55 (–6.7 to 1.59)		.23	

^a^All regressions included only one of the variables (sex; age group; education; place of residence; income; vocation; whether or not a participant has a family member, friend, or acquaintance who they know to have been infected with SARS-CoV-2) shown in the table and a binary indicator for each province (province-level fixed effects).

^b^N/A: not applicable.

## Discussion

On average, participants in our survey answered 21.4 (95% CI 21.3-21.4) out of 25 questions correctly. Higher knowledge scores were associated with being middle-aged, higher household income, male sex, and living in urban areas. Knowledge about prevention methods, common misconceptions, and transmission modes of SARS-CoV-2 infection did not vary markedly across provinces. However, knowledge about appropriate measures upon the appearance of suspicious symptoms showed a decreasing trend from the eastern wealthier coastal provinces to the western less wealthy inland provinces.

A significant proportion of participants in all provinces held misconceptions about prevention methods and recommended health care–seeking behaviors, such as beliefs that regular nasal saline rinses or gargling mouthwash are effective at preventing a SARS-CoV-2 infection. Moreover, 10.0% (95% CI 9.4%-10.6%) of participants reported that, if they had symptoms suggestive of a possible SARS-CoV-2 infection, they would continue with their daily routine. It is important for these false and potentially dangerous beliefs to be addressed in public health campaigns. These findings could inform campaigns focused on the dissemination of misinformation on the internet and on social media, both of which have been identified as platforms to circulate such misinformation [19]. It may also be useful to inform Chinese health care professionals about common misconceptions held by the public so that these misconceptions can be addressed in consultations.

Although the majority of participants demonstrated a high proportion of correct responses to knowledge questions, there was nonetheless important variation in knowledge by knowledge domain. Questions that assessed knowledge of who is at increased risk of severe COVID-19 and common symptoms of SARS-CoV-2 infection were answered correctly over 90% of the time. Questions about how SARS-CoV-2 is primarily transmitted and what precautions are effective against transmission had correct response rates between 81% and 84%. The areas of lowest knowledge were identifying ineffective measures for, or misconceptions about, prevention of SARS-CoV-2 transmission and recommended care-seeking behavior when developing a fever or new persistent cough. This suggests that public health campaigns in China can most efficiently improve the public’s knowledge about COVID-19 by dispelling misconceptions about ineffective prevention measures and developing clear, consistent, and widely distributed instructions on what individuals should do if they develop symptoms suggestive of COVID-19.

Our study results are comparable with those of studies from other countries that assessed knowledge and perceptions of COVID-19. In comparison to a similar survey in the United States and the United Kingdom [9], the majority of the results were similar across the three countries. However, we also observed differences in the beliefs about the effectiveness of masks between these countries. Nearly nine-tenths of Chinese participants reported to believe wearing a face mask was effective in preventing a SARS-CoV-2 infection, whereas only about 40% and 30% of the US and UK participants, respectively, expressed this belief, suggesting that Chinese participants were more likely to accept and select masks as a prevention measure against infections. This comparison is limited, however, as it is important to note that beliefs in the effectiveness of mask wearing in the United States and the United Kingdom have likely increased since that survey was conducted in February-March 2020. In Australia, an online survey undertaken between March 18-24, 2020, found that understanding and adoption of hygiene-related behaviors was high [20]. Surveys conducted in Nepal, Egypt, and Malaysia found that participants had good general knowledge about COVID-19, including modes of transmission and recommended prevention measures [21]. However, several misconceptions were common, such as avoidance of eating poultry and meat to reduce the chance of SARS-CoV-2 infection or that wearing masks in public does not reduce infections [21-23]. The prevalence of misinformation and misconceptions about COVID-19 worldwide underscores our recommendation that public health authorities should continue campaigns that dispel widespread misinformation and focus on consistent messaging about the importance of following a combination of evidence-based infection prevention methods and behaviors.

A key strength of our study is that we sampled a large number of individuals across China’s provinces, allowing us to assess how knowledge and perceptions vary across regions, and specifically to compare perceptions in provinces that have been less impacted by COVID-19 to those in more impacted areas like Hubei, the most severely affected province in China so far.

This study, however, also has several limitations. First, participants were selected from a pool of adults who were registered with KuRunData and who agreed to take the survey. It is possible that these individuals differ in important ways from the general population of China, as ability and willingness to register with an online survey company and participate in this survey might be related to literacy, education level, and perspectives on or experience with COVID-19, to name a few. Second, an important demographic limitation is the relatively small number of older adults in our sample. Although we used sampling weights to adjust for this pattern, our estimate for the oldest age groups had a small sample size and thus a large degree of uncertainty. Third, as answers were self-reported responses to hypothetical situations, they might not reflect what individuals would do in reality. Similarly, social desirability bias could have influenced participants to answer in ways that they thought they should, rather than how they truly felt. Finally, it must be noted that this survey was administered between May 8 and June 8, 2020, and consequently reflects knowledge and perceptions at that time.

In conclusion, although general knowledge about COVID-19 was comparatively high across China, a substantial proportion of the population was found to hold important misconceptions about some prevention methods and recommended health care–seeking behaviors. Chinese policy makers should address this misinformation, as it will be important that people have accurate knowledge and practice correct prevention measures to avoid a resurgence of infections.

## Appendix

Multimedia Appendix 1 Supplementary material.
