# Correction: Knowledge About COVID-19 Among Adults in China: Cross-sectional Online Survey

**DOI:** 10.2196/30100

**Published:** 2021-05-12

**Authors:** Fengyun Yu, Pascal Geldsetzer, Anne Meierkord, Juntao Yang, Qiushi Chen, Lirui Jiao, Nadeem E Abou-Arraj, An Pan, Chen Wang, Till Bärnighausen, Simiao Chen

**Affiliations:** 1 Department of Industrial Engineering Tsinghua University Beijing China; 2 Heidelberg Institute of Global Health Faculty of Medicine and University Hospital Heidelberg University Heidelberg Germany; 3 Division of Primary Care and Population Health Department of Medicine Stanford University School of Medicine Stanford, CA United States; 4 Faculty of Medicine University of Southampton Southampton United Kingdom; 5 State Key Laboratory of Medical Molecular Biology Institute of Basic Medical Sciences Chinese Academy of Medical Sciences and Peking Union Medical College Beijing China; 6 The Harold and Inge Marcus Department of Industrial and Manufacturing Engineering The Pennsylvania State University University Park, PA United States; 7 Reed College Portland, OR United States; 8 Department of Medicine Stanford University School of Medicine Stanford, CA United States; 9 School of Public Health Tongji Medical College Huazhong University of Science and Technology Wuhan China; 10 Chinese Academy of Medical Sciences and Peking Union Medical College Beijing China; 11 National Clinical Research Center for Respiratory Diseases Beijing China; 12 Department of Pulmonary and Critical Care Medicine Center of Respiratory Medicine China–Japan Friendship Hospital Beijing China

In “Knowledge About COVID-19 Among Adults in China: Cross-sectional Online Survey” (J Med Internet Res 2021;23(4):e26940) the authors noted three errors.

In the originally published manuscript, authors Chen Wang, Till Bärnighausen, and Simiao Chen were noted as having contributed equally to the manuscript.

This has been corrected to note that authors Fengyun Yu, Pascal Geldsetzer, Chen Wang, Till Bärnighausen, and Simiao Chen all contributed equally to the manuscript.

Additionally, the following figures have been revised with minor changes in scale and legend title.

- Figure 1: Map showing the mean overall knowledge score by province.
- Supplementary Figure A3: Map showing the mean overall knowledge score by province when excluding participants who reported looking up an answer online (Multimedia Appendix 1).

**Figure 1:**
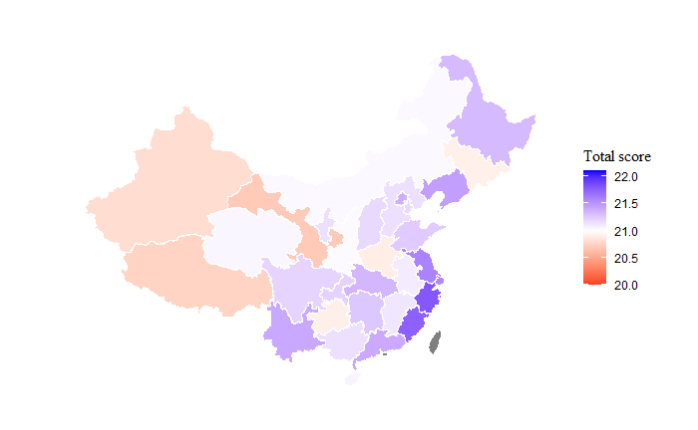
Map showing the mean overall knowledge score by province.

The correction will appear in the online version of the paper on the JMIR Publications website on May 12, 2021, together with the publication of this correction notice. Because this was made after submission to PubMed, PubMed Central, and other full-text repositories, the corrected article has also been resubmitted to those repositories.

## Appendix

Multimedia Appendix 1 Supplementary material.

