# Supplementary material for: Knowledge About COVID-19 Among Adults in China: Cross-sectional Online Survey
Source: J Med Internet Res. 2021 Apr 29;23(4):e26940. doi: 10.2196/26940 (PMC8086781; doi:10.2196/26940)
Supplement: Multimedia Appendix 1 [file jmir_v23i4e26940_app1.docx]

**Supplementary material for “Knowledge about Coronavirus Disease 2019 among adults in China: A cross-sectional online survey”**

**Figure A1. Time taken to complete the questionnaire in seconds**


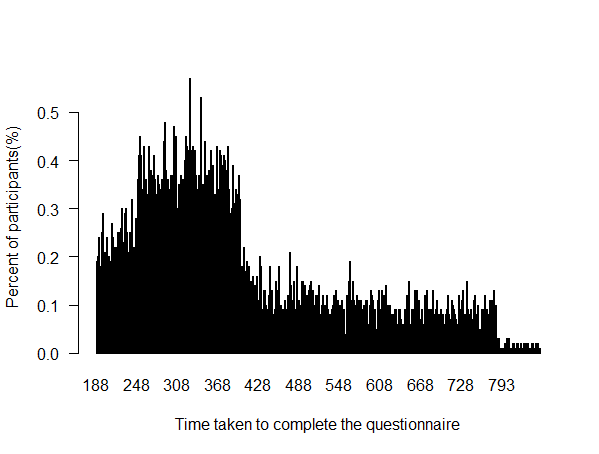


**Figure A2. Distribution of the overall knowledge score**


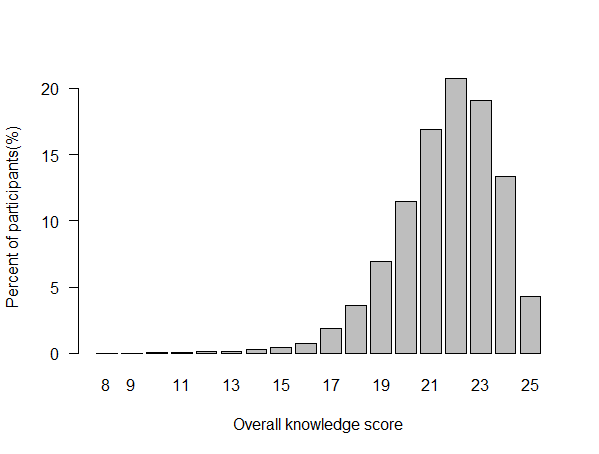


**Table A1. Summary of survey findings when excluding participants who reported looking up an answer online**^1^

^1^ Participants who self-reported looking up the answer to a question online were excluded from the analysis for that particular question only (as opposed to exclusion from the analysis for all questions).

^*^ This column shows how response options are grouped to summarize categorical variables into a dichotomous measure.

^†^  For dichotomous outcomes, data are expressed as percentage with correct response (95% confidence interval). For continuous outcomes, data are expressed as median (interquartile range).

| **Survey Question** | **Response*** | **Proportion or median estimate**^†^ |
| --- | --- | --- |
| **Perceived risk of death from COVID-19** |  |  |
| "What percent of individuals infected with the new coronavirus experience a fatal disease course?" | Continuous variable | 3.3% (IQR: 1.1%-3.8%) |
| "When they have been infected, what age groups are most likely to die from the illness caused by the new coronavirus?" | Children,  Young adults,  Older adults | 21.9% (95% CI: 20.9%-22.9%)  9.6% (95% CI: 8.9%-10.4%)  96.8% (95% CI: 96.4%-97.2%) |
| "Are those with other health problems more likely to die from an infection with the new coronavirus disease than those without any other health problems?" | Yes | 93.1% (95% CI: 92.4% - 93.7%) |
| "What percent of people who get infected with the common flu end up dying from the common flu?" | Continuous variable | 0.75% (IQR: 0.10% - 1.00%) |
| **Transmission of COVID-19** |  |  |
| "Only older adults can become infected with the new coronavirus." | False | 99.0% (95% CI: 98.8% - 99.2%) |
| "Is there currently a vaccine available that protects against infection with the new coronavirus?" | No | 80.3% (95% CI: 79.3% - 81.2%) |
| "Which of the following actions help prevent catching an infection with the new coronavirus?" | Selected all of the following: avoiding touching eyes, nose, mouth with unwashed hands, washing your hands, avoiding close physical contact with people who are sick  Selected at least one of the following: using a hand dryer, regularly rinsing your nose with saline, taking antibiotics, and gargling mouthwash. | 87.0% (95% CI: 86.1% - 87.8%)  66.1% (95% CI: 64.9% - 67.2%) |
| "Consistently wearing a face mask is highly effective in protecting you from getting infected with the new coronavirus." | True | 89.2% (95% CI: 88.4% - 89.9%) |
| "What is the main way in which people are currently getting infected with the new coronavirus?" | Droplets of saliva that land in the mouths or noses of people who are nearby when an infected person sneezes or coughs | 85.6% (95% CI: 84.7% - 86.4%) |
| **Symptoms of COVID-19 and recommended health care-seeking behavior** |  |  |
| “What are the common signs or symptoms of an infection with the new coronavirus?” | Nose bleeds,  Cough,  Fever,  Skin rash,  Constipation,  Shortness of breath,  Frequent urination | 9.1% (95% CI: 8.4%-9.8%)  98.5% (95% CI: 98.2% - 98.8%)  99.7% (95% CI: 99.5% - 99.8%)  7.4% (95% CI: 6.8% - 8.0%)  6.0% (95% CI: 5.4% - 6.6%)  91.9% (95% CI: 91.2% - 92.6%)  3.3% (95% CI: 2.9% - 3.8%) |
| “If you have a fever or cough and recently visited China, or spent time with someone who did, what would be the best course of action?” | Go directly to a hospital,  Call the official hotline,  Continue my daily routine | 35.3% (95% CI: 34.2% - 36.5%)  39.3% (95% CI: 38.1% - 40.5%)  11.1% (95% CI: 10.4% - 11.9%) |

**Table A2. Covariate-unadjusted regression results when excluding participants who reported looking up an answer online**^1^

| **Characteristics** | **Absolute difference in the number of questions that were answered correctly (95% CI)** | **p value** |
| --- | --- | --- |
| ***Sex*** | | |
| Male | 0 (ref.) |  |
| Female | -0.46 (-0.57 - -0.35) | <0.001 |
| ***Age*** | | |
| 18-19 years | 0 (ref.) |  |
| 20-29 years | 0.83 (0.57 - 1.1) | <0.001 |
| 30-39 years | 1.32 (1.05 - 1.59) | <0.001 |
| 40-49 years | 1.27 (1.01 - 1.53) | <0.001 |
| 50-59 years | 0.79 (0.52 - 1.05) | <0.001 |
| >60 years | 0.68 (0.42 - 0.95) | <0.001 |
| ***Education*** | | |
| Never been to school | 0 (ref.) |  |
| Elementary school | -0.16 (-0.58 - 0.25) | 0.444 |
| Middle school | -0.2 (-0.52 - 0.13) | 0.245 |
| High school / Technical secondary school | -0.3 (-0.61 - 0.01) | 0.058 |
| College / Undergraduate | 0.48 (0.17 - 0.78) | 0.002 |
| Graduate and Above | 0.42 (-0.07 - 0.91) | 0.093 |
| ***Place of residence*** | | |
| Rural | 0 (ref.) |  |
| Urban | 1.47 (1.34 - 1.60) | <0.001 |
| ***Work as a healthcare provider*** | | |
| Not | 0 (ref.) |  |
| Nurse | 0.23 (-0.56 - 1.01) | 0.576 |
| Physician | 0.67 (0.11 - 1.24) | 0.020 |
| Community health worker | -0.39 (-1.17 - 0.4) | 0.332 |
| Pharmacist | 1.14 (0.14 - 2.14) | 0.026 |
| Other healthcare provider | -1.12 (-1.96 - -0.27) | 0.010 |
| ***Annual household income (RMB)*** | | |
| < 30,000 | 0 (ref.) |  |
| 30,000 - 59,999 | 0.8 (0.41 - 1.19) | <0.001 |
| 60,000 - 89,999 | 0.87 (0.49 - 1.25) | <0.001 |
| 90,000 - 119,999 | 1.26 (0.88 - 1.64) | <0.001 |
| 120,000 - 149,999 | 1.67 (1.28 - 2.07) | <0.001 |
| 150,000 - 199,999 | 1.64 (1.24 - 2.05) | <0.001 |
| ≥ 200,000 | 1.76 (1.34 - 2.19) | <0.001 |
| ***PHQ-9 score*** | | |
| 0-4 | 0 (ref.) |  |
| 5-9 | 0.09 (-0.04 - 0.21) | 0.179 |
| >9 | -0.3 (-0.58 - -0.02) | 0.034 |
| ***Knows someone with a confirmed SARS-CoV-2 infection*** | | |
| Self | 2.66 (1.46 - 3.86) | <0.001 |
| Neighbors | -1.83 (-2.51 - -1.16) | <0.001 |
| Coworkers | 0.41 (-1.2 - 2.03) | 0.618 |
| Friend | -0.04 (-0.91 - 0.84) | 0.932 |

^1^ Included only one of the variables (sex, age group, education, place of residence, income, vocation, PHQ-9 score, whether or not a participant has a family member, friend, or acquaintance who they know to have been infected with SARS-CoV-2) shown in the table and a binary indicator for each province (province-level fixed effects).

**Table A3. Regression results when adjusting for age group and sex**^1^

| **Characteristics** | **Absolute difference in the number of questions that were answered correctly (95% CI)** | **p value** |
| --- | --- | --- |
| ***Sex*** | | |
| Male | 0 (ref.) |  |
| Female | -0.34 (-0.44 - -0.24) | <0.001 |
| ***Age*** | | |
| 18-19 years | 0 (ref.) |  |
| 20-29 years | 0.63 (0.41 - 0.85) | <0.001 |
| 30-39 years | 0.97 (0.74 - 1.19) | <0.001 |
| 40-49 years | 1.07 (0.85 - 1.29) | <0.001 |
| 50-59 years | 0.61 (0.38 - 0.83) | <0.001 |
| >60 years | 0.69 (0.47 - 0.91) | <0.001 |
| ***Education*** | | |
| Never been to school | 0 (ref.) |  |
| Elementary school | -0.36 (-1.15 - 0.42) | 0.365 |
| Middle school | -0.78 (-1.66 - 0.11) | 0.086 |
| High school / Technical secondary school | -1.25 (-1.87 - -0.64) | <0.001 |
| College / Undergraduate | -0.01 (-1.51 - 1.49) | 0.991 |
| Graduate and Above | -0.7 (-1.28 - -0.12) | 0.018 |
| ***Place of residence*** | | |
| Rural | 0 (ref.) |  |
| Urban | 1.25 (1.14 - 1.36) | <0.001 |
| ***Work as a healthcare provider*** | | |
| Not | 0 (ref.) |  |
| Nurse | -0.33 (-1.14 - 0.48) | 0.424 |
| Physician | -0.86 (-1.71 - -0.02) | 0.045 |
| Community health worker | -1.27 (-1.87 - -0.68) | <0.001 |
| Pharmacist | -0.1 (-1.55 - 1.35) | 0.892 |
| Other healthcare provider | -0.41 (-0.96 - 0.14) | 0.145 |
| ***Annual household income (RMB)*** | | |
| < 30,000 | 0 (ref.) |  |
| 30,000 - 59,999 | 0.61 (0.31 - 0.92) | <0.001 |
| 60,000 - 89,999 | 0.7 (0.4 - 1) | <0.001 |
| 90,000 - 119,999 | 1.02 (0.72 - 1.31) | <0.001 |
| 120,000 - 149,999 | 1.3 (0.99 - 1.61) | <0.001 |
| 150,000 - 199,999 | 1.32 (0.99 - 1.64) | <0.001 |
| ≥ 200,000 | 1.27 (0.92 - 1.63) | <0.001 |
| ***PHQ-9 score*** | | |
| 0-4 | 0 (ref.) |  |
| 5-9 | 0.1 (-0.01 - 0.21) | 0.063 |
| >9 | -0.5 (-0.77 - -0.24) | <0.001 |
| ***Knows someone with a confirmed SARS-CoV-2 infection*** | | |
| Self | -0.79 (-3.58 - 2) | 0.581 |
| Family | -0.74 (-6.16 - 4.69) | 0.790 |
| Neighbors | -1.4 (-3.32 - 0.52) | 0.154 |
| Coworkers | 0.81 (-0.61 - 2.22) | 0.266 |
| Friend | 0.82 (0.55 - 1.08) | <0.001 |

^1^ The regressions included one of the independent variables (education, place of residence, income, vocation, PHQ-9 score, whether or not a participant has a family member, friend, or acquaintance who they know to have been infected with SARS-CoV-2) shown in the table, a binary indicator for each province (province-level fixed effects), age group, and sex. The regression for age group was adjusted for sex and province-level fixed effects, and the regression for sex for age group and province-level fixed effects.

**Figure A3. Map showing the mean overall knowledge score by province when excluding participants who reported looking up an answer online**^1,2,3^


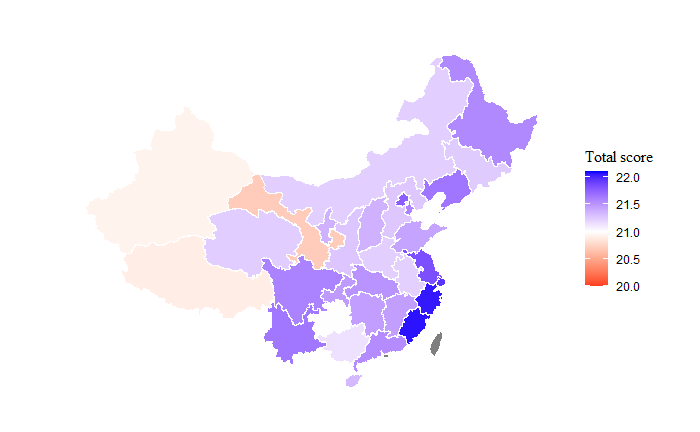


^1^ Participants who self-reported looking up the answer to a question online were excluded from the analysis for that particular question only (as opposed to exclusion from the analysis for all questions).

^2^ The minimum score was 0 and the maximum score was 25.

^3^ Taiwan, Hongkong, and Macao are shown in grey.

**Figure A4. Proportion of the population by province with correct responses to questions about prevention methods (a), common misconceptions (b), transmission channels (c), and recommended actions after infection (d), when excluding participants who reported looking up an answer online^1,2,3,4,*^**


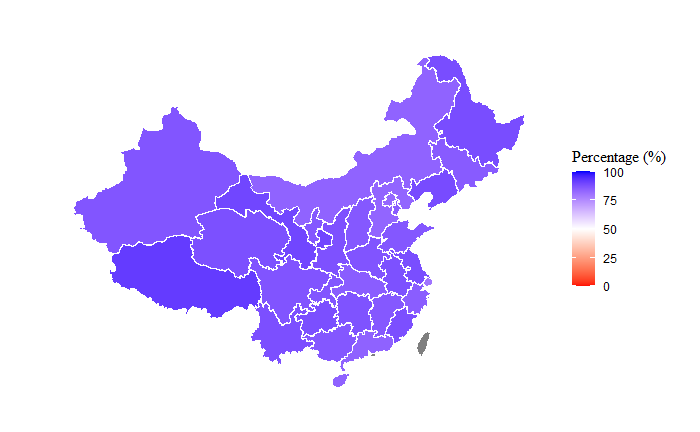

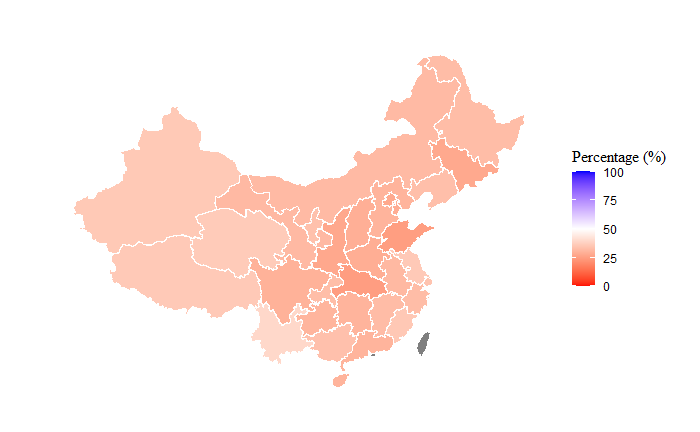


**(a) (b)**


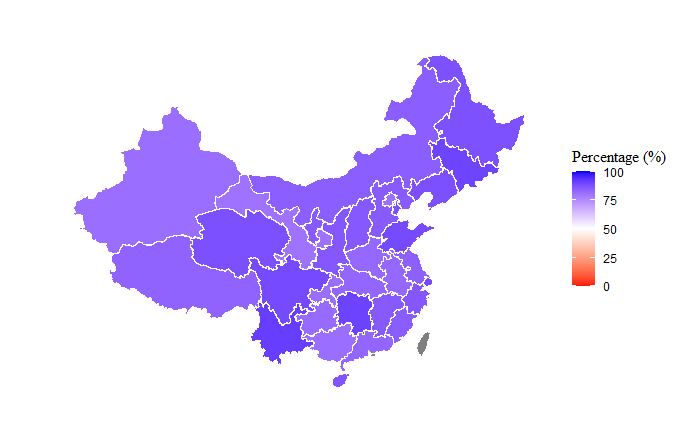

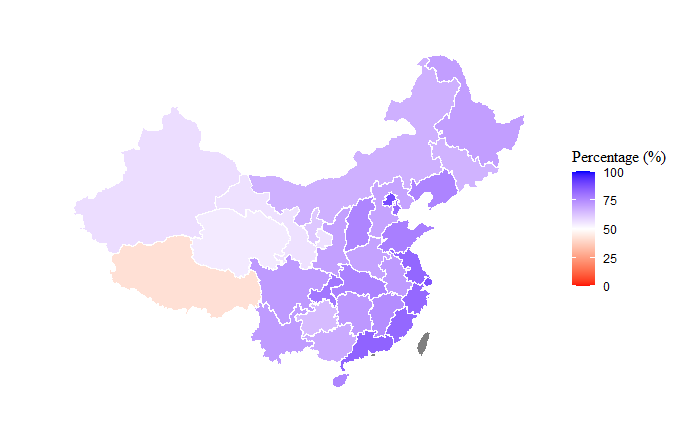


**(c) (d)**

^1^ Panel (a) shows the proportion of participants who believed all of the following measures are helpful for preventing a SARS-CoV-2 infection: avoiding touching eyes, nose, and mouth with unwashed hands; washing your hands; avoiding close physical contact with people who are sick.

^2^ Panel (b) shows the proportion of participants who did **not** select any of the following as being helpful in preventing a SARS-CoV-2 infection: using a hand dryer, regularly rinsing your nose with saline, taking antibiotics, and gargling mouthwash.

^3^ Panel (c) shows the proportion of participants who believed people are mainly infected with SARS-CoV-2 through droplets of saliva from infected persons.

^4^ Panel (d) shows the proportion of participants who reported that they would go directly to a hospital or call the official hotline if they had a fever and new persistent cough.

^*^ Taiwan province, Hongkong and Macao are shown in grey.

**Text A1. Questionnaire text in English version**

**Introduction**

A new virus (of the coronavirus family) emerged in Wuhan, China, in 2019. We will refer to this virus from now on simply as “the new coronavirus”. We would love to find out what the general public in China knows about this new virus.

For this research to be helpful to public health efforts, it is important that you refrain from looking up any answers online. A lot of aspects about this virus and the illness it causes are still unknown, so much of the time there is no correct answer. Please just give it your best guess. All you need to do to receive your payment is to fill out the answers to the best of your knowledge.

Perceived risk of death from COVID-9

- Q1: What percent of people who get infected with the new coronavirus die from this infection? No one knows the correct answer to this, so please just give it your best guess.(*Please enter a number between 0 and 100. Decimals are allowed.*)
- Q2: When people have been infected, what age groups are most likely to die from the illness caused by the new coronavirus? (*Please select all options that you think are correct.*)

□ Children

□ Yound adults

□ Older adults

- Q3: Are those with other health problems more likely to die from an infection with the new coronavirus disease than those without any other health problems?
- □ Yes
- □ No
- Q4: What percent of people who get infected with the common flu end up dying from the common flu? (*Please enter a number between 0 and 100. Decimals are allowed.*)

**Transmission of the new coronavirus**

- Q5: Only older adults can become infected with the new coronavirus.
- □ True
- □ False
- Q6: Is there currently a vaccine available that protects against infection with the new coronavirus?
- □ Yes
- □ No
- Q7: Which of the following actions help prevent catching an infection with the new coronavirus? (*Please select 'True' or 'False' for each option.*)

|  | True | False |
| --- | --- | --- |
| Wear a face mask |  |  |
| Getting a vaccination against pneumonia |  |  |
| Gargling mouthwash |  |  |
| Washing your hands |  |  |
| Eating garlic |  |  |
| Avoid close contact with people who are sick |  |  |
| Taking antibiotics |  |  |
| Using a hand dryer |  |  |
| Putting sesame oil on your skin |  |  |
| Avoiding touching your eyes, nose, and mouth with unwashed hands |  |  |
| Regularly rinsing your nose with saline |  |  |

- Q8: Is the following statement true or false? Consistently wearing a face mask is highly effective in protecting you from getting infected with the new coronavirus.

For the purpose of this question, "highly effective" is defined as reducing your risk of getting infected by >95% and a "face mask" is a common medical mask.

- □ True
- □ False
- Q9: What is the main way in which people are currently getting infected with the new coronavirus?

*Please select one response option only*.

- □ Eating or touching bats
- □ Fecal containminants in drinking water
- □ Unhygienic preparation of food
- □ Sexual intercourse or sharing of needles for drug use
- □ Mosquito bites
- □ Droplets of saliva that land in the mouths or noses of people who are nearby when an infected person sneezes or coughs
- □ Eating undercooked meat products
- □ Directly coming into touch with someone's bodily fluids like blood, vomit, or sweat
- □ Snake bites or touching snakes
- Q10: Approximately how far do you think the new coronavirus can travel through the air to transmit the infection from one person to another?
  *Please give the number of meters. Decimals are allowed.*

**Recognizing and acting upon an infection**

- Q11: What are common signs or symptoms of an infection with the new coronavirus? (*Please select ‘True or ‘False’ for each option.*)

|  | True | False |
| --- | --- | --- |
| Nose bleeds |  |  |
| Cough |  |  |
| Fever |  |  |
| Skin rash |  |  |
| Constipation |  |  |
| Shortness of breath |  |  |
| Frequent urination |  |  |

- Q12: If you had a fever and new persistent cough that started today, what would you do? (*Please select one response option only*)
- □ Seek help from friends
- □ Go directly to a hospital—such as a designated hospital, temporary hospital, or Fangcang shelter hospital
- □ Stay at home, rest, and decide whether to take some medications
- □ Call the official hotline for the new coronavirus disease, or contact your community health worker or other official contact person
- □ Continue with your daily routine (go outside, travel, or meet friends) and see if you feel better after a few days
- □ Seek help privately from doctors who claim they have cured many patients infected with new coronavirus
- □ Go to a hotel to self-quarantine, rest, and decide whether to take some medications
- Q13: In the past 12 months, have you experienced the following conditions for two weeks or longer?

| No. | Conditions | Never | Several days | More than half of the time | Almost everyday |
| --- | --- | --- | --- | --- | --- |
| 1 | Unmotivated or uninterested in doing things | 0 | 1 | 2 | 3 |
| 2 | Feeling down, depressed or hopeless | 0 | 1 | 2 | 3 |
| 3 | Having difficulty falling asleep; sleeping restless or oversleeping | 0 | 1 | 2 | 3 |
| 4 | Feeling tired or inactive | 0 | 1 | 2 | 3 |
| 5 | Loss of appetite or eating too much | 0 | 1 | 2 | 3 |
| 6 | Feeling bad or like a failure, or is disappointed with myself and my family | 0 | 1 | 2 | 3 |
| 7 | Having difficulty focusing on things like reading a newspaper or watching TV, etc. | 0 | 1 | 2 | 3 |
| 8 | Move or speak so slowly that other people have noticed it; or oppositely—become more irritable or figdet around | 0 | 1 | 2 | 3 |
| 9 | I ’d rather die or hurt myself in some way. | 0 | 1 | 2 | 3 |

- Q14: It is natural to be tempted to look up the answer to a question, especially when it’s only a click away. For approximately how many of the questions above did you first look up the answer on Baidu or somewhere else before responding? The answer to this question will not affect your payment in any way. (Please enter a number below. Decimals are not allowed)

Display This Question:

If “If It is natural to be tempted to look up the answer to a question, especially when it’s only a click away. For approximately how many of the questions above did you first look up the answer on Baidu...” Text Response Is Greater Than 0

- Q15: For which question(s) did you look up the answer on Baidu or somewhere else before responding? Again, the answer to this question will not affect your payment in any way. (*Please select all questions that apply*)
- [We list questions here that we asked]

**Sociodemographics**

- Q16: Have you ever been diagnosed with the new coronavirus disease?
- □ Yes
- □ No
- Q17: If yes to Q16, when were you diagnosed?
- Date:
- Q18 Has anyone in your family, neighbors, coworkers, friends or other people you know been diagnosed with the new coronavirus disease?
- □ Family member
- □ Friend
- □ Neighbors
- □ Coworkers
- □ Other people I know, please specify:
- Q19: What is yout age? (*Please enter a number. Decimals are not allowed.*)
- Q20: What is your gender?
- □ Male
- □ Female
- Q21a In which province do you currently reside?

| 1 | Beijing |
| --- | --- |
| 2 | Shanghai |
| 3 | Tianjin |
| 4 | Chongqing |
| 5 | Liaoning |
| 6 | Jilin |
| 7 | Heilongjiang |
| 8 | Hebei |
| 9 | Shanxi |
| 10 | Neimengol |
| 11 | Jiangsu |
| 12 | Zhejiang |
| 13 | Anhui |
| 14 | Fujian |
| 15 | Jiangxi |
| 16 | Shandong |
| 17 | Henan |
| 18 | Hubei |
| 19 | Hunan |
| 20 | Guangdong |
| 21 | Guangxi |
| 22 | Henan |
| 23 | Sichuan |
| 24 | Guizhou |
| 25 | Yunnan |
| 26 | Tibet |
| 27 | Shaanxi |
| 28 | Gansu |
| 29 | Qinghai |
| 30 | Ningxia |
| 31 | Xinjiang |

- Q21D: Where do you live?
- □ Urban
- □ Rural
- Q22: What is the highest degree or level of school you have completed? (If you’re currently enrolled in school, please indicate the highest degree you have received.)
- □ Never been to school
- □ Elementary school
- □ Middle school
- □ High school / Technical secondary school
- □ College / Undergraduate
- □ Graduate and above
- Q23: Are you a healthcare provider, such as a nurse, physician, community health worker, or pharmacist?
- □ No, I'm not a healthcare provider
- □ Nurse
- □ Physician
- □ Community health worker
- □ Pharmacist
- □ Other healthcare provider; please specify:
- Q24: What is your ethnicity? (*Please select all options that apply*)
- □ Han
- □ Hui
- □ Zang
- □ Zhuang
- □ Man
- □ Other; please specify:

**Text A1. Questionnaire text in Chinese version**

**简介**

2019年，中国武汉出现了一种新型病毒（隶属于冠状病毒家族），我们将这种病毒简称为“新型冠状病毒”。我们希望调研中国公众对这种新病毒的认识。

为了使这项研究的结果对公共卫生工作有所帮助，请务必不要在线查找任何答案，这一点很重要。关于该病毒及其引起的疾病的很多方面仍然属于未知数，因此很多问题没有准确答案。请给出您的最佳猜测，您收到报酬后所需要做的就是尽您所能填写答案。

**感受到新型冠状病毒的死亡威胁**

- Q1:新冠状病毒感染者的死亡比例是多少？没有人知道准确的答案，请给出您的最佳猜测。（*请填写0-100之间的数字，允许填写小数*）
- Q2: 当人感染新冠肺炎后，哪个年龄组最有可能死于新型冠状病毒引起的疾病？(*请选择所有您认为正确的选项。*)

□ 儿童

□ 年轻人

□ 老年人

- Q3: 与没有其他健康问题的人相比，那些本身患有其他健康问题的人是否更有可能死于新型冠状病毒感染？
- □ 是
- □ 否
- Q4: 感染普通流感的人中最终死于普通流感的比例是多少？(*请填写0-100之间的数字，允许填写小数.*)

**新型冠状病毒的传播**

- Q5: 只有老年人才会感染新冠肺炎。
- □ 正确
- □ 错误
- Q6: 当前是否有可预防新冠肺炎感染的疫苗？
- □ 是
- □ 否
- Q7: 以下哪项行为有助于预防感染新冠肺炎？ (*请为每个选项选择“正确”或“错误”.*)

|  | 正确 | 错误 |
| --- | --- | --- |
| 戴口罩 |  |  |
| 接种预防肺炎的疫苗 |  |  |
| 漱口水漱口 |  |  |
| 洗手 |  |  |
| 吃大蒜 |  |  |
| 避免与感染者密切接触 |  |  |
| 服用抗生素 |  |  |
| 使用干手器 |  |  |
| 将芝麻油涂在皮肤上 |  |  |
| 避免用未洗的手触摸眼睛，鼻子和嘴巴 |  |  |
| 定期用盐水冲洗鼻子 |  |  |

- Q8: 以下陈述正确与否？ 始终如一地戴口罩能非常有效地保护您免受新冠肺炎感染。

在这个问题中，“非常有效”的定义是使感染的风险低于95％，“口罩”指的是常见的医用口罩。

- □ 正确
- □ 错误
- Q9: 当前人们感染新冠肺炎的主要途径是什么？

*请仅选择一个选项。*

- □ 食用或触碰蝙蝠
- □ 饮用水中的粪便污染物
- □ 准备食物时不卫生
- □ 性交或吸毒时共用针头
- □ 蚊虫叮咬
- □ 当感染打喷嚏或者咳嗽时，飞沫喷溅至附近人们的嘴或鼻子上
- □ 食用未煮熟的肉类产品
- □ 直接接触某人的体液，例如血液、呕吐物或汗水
- □ 被蛇咬到或触碰蛇
- Q10: 您认为新冠病毒可以在空中传播多远，使得感染从一个人传播至另一个人？（*请填写0-100之间的数字，允许填写小数.*）

**认识感染并采取行动**

- Q11: 新冠肺炎感染的常见体征或症状是什么？ (*请为每个选项选择“正确”或“错误”。*)

|  | 正确 | 错误 |
| --- | --- | --- |
| 流鼻血 |  |  |
| 咳嗽 |  |  |
| 发烧 |  |  |
| 皮疹 |  |  |
| 便秘 |  |  |
| 呼吸急促 |  |  |
| 尿频 |  |  |

- Q12: 如果您从今天开始出现发烧和持续咳嗽的症状，您该怎么办？ (*请仅选择一个选项*)
- □ 向朋友寻求帮助
- □ 直接去医院，例如指定医院、临时医院或方舱医院
- □ 留在家中休息，并决定是否服药
- □ 致电新冠肺炎的官方热线，或联系您的社区卫生工作者或其他官方联系人
- □ 继续进行日常活动（外出、旅行或与朋友见面），看几天后状况是否有所好转
- □ 私下寻求医生（声明自己已经治愈了许多新冠肺炎患者）的帮助
- □ 去酒店自我隔离、休息，然后决定是否要服药
- Q13: 在答题时想查找答案是很自然的事情，尤其是当一个单击即可的时候。在答题前，您先在百度或其他地方查询了大概几个上面的问题？这个问题的答案不会以任何方式影响您的酬劳。 (*请填写0-100之间的数字，不允许填写小数*)

显示此问题：

如果是“在答题时想查找答案是很自然的事情，尤其是当一个单击即可的时候。在答题前，您先在百度或其他地方查询了大概几个上面的问题？……”的答案大于0

- Q14: 在答题之前，您是针对哪个问题在百度或其他地方查询了答案？同样，此问题的答案不会以任何方式影响您的酬劳。 (*请选择所有符合的选项*)
- [我们在此列出了我们提出的所有问题]

**社会人口学**

- Q15: 您是否曾被诊断出患有新冠肺炎？
- □ 是
- □ 否
- Q16: 如果第15题的答案是“是”，您是何时被确诊的？
- 日期:
- Q17 您的家庭成员、邻居、同事、朋友或您认识的其他人中是否有人被诊断出患有新冠肺炎？
- □ 家庭成员
- □ 朋友
- □ 邻居
- □ 同事
- □ 我认识的其他人，请说明：
- Q18: 您的年龄是？(*请填写数字，不允许填写小数*)
- Q19: 您的性别是？
- □ 男性
- □ 女性
- Q20: 您现在居住在哪个省份？

| 1 | 北京 |
| --- | --- |
| 2 | 上海 |
| 3 | 天津 |
| 4 | 重庆 |
| 5 | 辽宁 |
| 6 | 吉林 |
| 7 | 黑龙江 |
| 8 | 河北 |
| 9 | 山西 |
| 10 | 内蒙古 |
| 11 | 江苏 |
| 12 | 浙江 |
| 13 | 安徽 |
| 14 | 福建 |
| 15 | 江西 |
| 16 | 山东 |
| 17 | 河南 |
| 18 | 湖北 |
| 19 | 湖南 |
| 20 | 广东 |
| 21 | 广西 |
| 22 | 海南 |
| 23 | 四川 |
| 24 | 贵州 |
| 25 | 云南 |
| 26 | 西藏 |
| 27 | 陕西 |
| 28 | 甘肃 |
| 29 | 青海 |
| 30 | 宁夏 |
| 31 | 新疆 |

- Q21: 请问您居住在农村还是城镇?
- □ 城镇
- □ 农村
- Q22: 您的最高学历是？（如果现在依然在校读书，请选择您已念完的最高学历）
- □ 没上过学
- □ 小学
- □ 初中
- □ 高中/中专
- □ 大专/本科
- □ 研究生及以上
- Q23: 您是医疗保健行业从业人员吗？例如护士、医师、社区卫生工作者或药剂师？
- □ 不，我不是医疗保健行业从业人员
- □ 护士
- □ 医师
- □ 社区卫生工作者
- □ 药剂师
- □ 其他医疗保健行业从业人员，请说明：:
- Q24: 您的民族是？(*请选择所有符合的选项*)
- □ 汉族
- □ 回族
- □ 藏族
- □ 壮族
- □ 满族
- □ 其他，请说明
